# Ultraviolet light spectroscopic characterization of ibuprofen acid aggregation in deionized water

**DOI:** 10.1016/j.heliyon.2023.e21260

**Published:** 2023-10-20

**Authors:** Gregorio Marbán, Amparo Fernández-Pérez, Sonia Álvarez-García

**Affiliations:** a Instituto de Ciencia y Tecnología del Carbono (INCAR-CSIC), c/Francisco Pintado Fe 26, 33011, Oviedo, Spain; b Departamento de Ingeniería Química y Tecnología del Medio Ambiente, Facultad de Químicas, Universidad de Oviedo, c/Julián Clavería 8, 33006, Oviedo, Spain

**Keywords:** Ibuprofen acid, Self-assembly, Aggregation equilibrium, UV–Visible spectroscopy, Beer-Lambert law

## Abstract

This work provides a description of the aggregation equilibria of ibuprofen acid in deionized water at temperatures between 20 and 40 °C in the 0.1–20.1 ppm concentration range. For this goal, we have made use of UV–Visible spectroscopy. A calculation algorithm was developed to obtain the aggregate orders and thermodynamic parameters from the experimental absorbance values. Monomeric ibuprofen acid was found to be absent in water solutions. In addition to the dimer, two aggregates formed by 32 and 128 monomeric units were found to co-exist in solution at the highest concentration tested. A critical micelle concentration of 7.8 ppm was estimated for this system. The appearance of the first aggregate occurs when the pH drops below the *pK*_*a*_ value, which was determined to be 4.62. At higher ibuprofen concentrations, a sudden jump in the electrical conductivity coincides with the onset of formation of the second aggregate. A varied menu of alternatives is offered with respect to the calibration curve of ibuprofen in water, though the linear calibration of ibuprofen concentration with absorbance might be reasonably performed at 224 nm. Finally, the dissolution rate of the commercial ibuprofen used in this work was found to obey the Noyes-Whitney first order equation.

## 1 Introduction

Ibuprofen, (±)-(R,S)-2-(4-isobutylphenyl)-propionic acid, is a chiral 2-arylpropionic acid derivative nonsteroidal anti-inflammatory drug (NSAID) and is used for the management of mild to moderate pain and inflammation. Though S-(+)-ibuprofen is about 160 times more potent than R-(−)-ibuprofen in inhibiting prostaglandin synthesis in vitro, for economic reasons the typical commercial ibuprofen is sold as a racemic or equal mixture of (R) and (S) mirror-image enantiomers. For ease of reference, we will refer to this racemic mixture as Ibu-H, or simply as ibuprofen. Ibu-H exists as a cyclic hydrogen bonded dimer in a solid or liquid state [[1], [2], [3], [4], [5], [6]]. Since its pharmacological activity is assigned to the monocarboxyl groups of an ibuprofen monomer, it is beneficial to introduce ibuprofen into the body as a monomer, for instance as a sodium salt, for an improved performance due to a higher drug solubility [2]. The amphiphilic character of Ibu-H makes it slightly soluble in water at 25 °C, though it is soluble in most organic solvents. The literature provides variable values of aqueous solubility at 25 °C within the 40–80 ppm range (39.6 ppm, evaluated by potentiometric titration [7]; 56 ppm [8], or ∼80 ppm (at 27 °C) [9], determined by HPLC after 48 h of magnetic stirring; also ∼80 ppm evaluated by UV–Visible spectroscopy after 98 h [10]; and 45 ppm evaluated by HPLC after several days for equilibrium [11]). As mentioned above, due to its low aqueous solubility, the sodium salt of ibuprofen (Ibu-Na), which has water solubility values over 1500 mM or 3.4 × 10^5^ ppm [12], is typically employed in the preparation of ibuprofen delivery systems [13]. Therefore, due attention is rightly paid to the development and performance of these systems [[14], [15], [16], [17]], which include surfactants that form micelles able to disperse the Ibu-Na molecules [13]. These micellar systems and their aggregation properties in the presence of the sodium salt of ibuprofen have been thoroughly analysed [13,[18], [19], [20]]. Even the self-assembly of Ibu-Na alone in water has been profusely studied [12,19,[21], [22], [23], [24]]. These works show that Ibu-Na has a critical micelle concentration of 180 mM (∼4.1 × 10^4^ ppm) [12,19,21], with a micelle aggregate number of either ∼40–48 [19,[21], [22], [23]], obtained with external fluorescent probes such as pyrene, or 6–20 [24], resulting from using direct back-face transmission steady-state fluorescence.

However, there are hardly any studies on the self-aggregation of aqueous ibuprofen in its acid form (Ibu-H) in the absence of electrolytes or other solubility enhancers, apart from the basic solubility studies previously mentioned [[7], [8], [9], [10], [11]]. The lack of studies on this subject is at least surprising, given the increasing importance of the aqueous ibuprofen disposal as a potential source of ecotoxicological risk [25,26], a situation aggravated by the huge increase in the use of generic drugs during the COVID-19 pandemic [27], which has generated a considerable number of works on ibuprofen decomposition [[28], [29], [30], [31], [32], [33]]. Therefore, the self-assembly of Ibu-H in pure water is an essential issue that still remains to be explored. As commented above, in a solid or liquid state the monomeric ibuprofen is known to self-associate through hydrogen bonds of its carboxylic groups resulting in its dimerization into a N-shaped dimer and a U-shaped dimer [[3], [4], [5], [6]]. The purpose of this work is to characterize the aggregation of Ibu-H in deionized water, beginning by determining which is the least aggregated species in the aqueous medium: dimer or monomer. For this goal, we will make use of UV–Visible spectroscopy. This technique is typically employed to evaluate ibuprofen concentrations in solution (up to 500 ppm in buffered solutions [[34], [35], [36], [37]], or in the 5–25 ppm range in pure water [38]). It is also known that the self-assembly of molecules with a low level of aggregation (*i.e.*, methylene blue) can be successfully studied by UV–Visible spectroscopy [39,40]. However, this technique is less often employed in the characterization of systems with a high aggregation degree [41], for example surfactants, for which other techniques such as conductometry, tensiometry, fluorimetry and potenciometry are generally preferred in order to evaluate the critical micelle concentration (or, more correctly, the critical aggregation concentration [42]) and the micelle aggregate number [43,44]. In this sense, Sosnik urges caution when using UV–Visible spectroscopy because in his view the absorption peaks associated with the aggregate not always comply with the Beer–Lambert law [42]. He supported this assertion by the works of Glisoni et al. [45], López-Nicolás and García-Carmona [46], and Fernández et al. [47]. However, in these works, and more specifically in the work by Fernández et al. [47], it seems clear that the Beer–Lambert law is certainly accomplished if/when it is applied to all the species in solution, including the aggregates, in order to evaluate their species-specific attenuation coefficients as well as their equilibrium constants of formation from the monomer aggregation. This exercise is based on the conviction that the aggregation equilibria fully comply with the mass action law [48], and the only difficulty is in dealing with high exponents (aggregate orders) that make the concentration terms in the equilibrium constants virtually infinite or zero, depending on the monomer concentration value. If an algorithm is found to tackle this issue with success, then the Beer–Lambert law may provide information which is much more meaningful that the mere critical parameters offered by other techniques. For instance, the quantification of the ratio between non-aggregated and aggregated species should be possible. In this work, an algorithm that allows the characterization of the aggregation of Ibu-H in deionized water has been developed. Said algorithm demonstrates the feasibility of using UV–Visible spectroscopy for such a task.

## 2 Experimental

Ibuprofen [(±)-(R,S)-2-(4-isobutylphenyl)-propionic acid; >99 %] was purchased from Sigma Aldrich. HPLC gradient grade methanol (J.T. Baker) and absolute ethanol (Emsure) were used as occasional solvents. All the aqueous solutions were prepared with deionized water. An aqueous stock solution of ibuprofen (20 ppm) was prepared in deionized water and subjected to magnetic stirring for three days, time during which the aggregation equilibria were attained (Figs. SI–1 in the *Supplementary Information* file). Then, analytical solutions were prepared by dilution and subjected to further stirring overnight prior analysis. The absorption spectra (190–300 nm at 0.2 nm step) of 14 different ibuprofen solutions [4.9 × 10^−7^ - 9.7 × 10^−5^ M (0.1–20.1 ppm)] were obtained at 20, 30 and 40 °C using an UV–Vis spectrometer (Shimazdu UV-2401PC) with UV quartz cuvettes of 17500 μL volume (5 cm path length) and 3500 μL volume (1 cm path length). The temperature of the cell was kept constant using a Lauda Alpha RA8 thermo-circulating bath. Every measure was repeated thrice. Baseline correction was made using deionized water (or the corresponding solvent when methanol or absolute ethanol were occasionally used). In no case was light absorption by the ibuprofen solutions at wavelength values over 300 nm detected. The electrical conductivity and pH of ibuprofen solutions in water at 20 °C were evaluated by the addition method in a SevenExcellence Professional Multi-Channel Meter (Mettler Toledo). For these tests, 5 mL of ibuprofen stock solution (20 ppm) was added to 50 mL water in a constant temperature bath, stirred up for 2 min and analysed. Ibuprofen addition was repeated at varying volumes of the stock solution (1–40 mL) until a final concentration of 16 ppm in the analysis beaker was reached.

## 3 Calculation algorithm

Application of the Beer–Lambert equation to the absorption spectrum of a mixture of light absorbing species in solution at a given wavelength yields [49]:

(1) $$A_{\lambda_{i},k,q}=\sum_{j=1}^{s}A_{\lambda_{i},k,q,j}=I_{F}L\sum_{j=1}^{s}\left(\varepsilon_{\lambda_{i},j}C_{k,q,j}\right)$$

in this equation *A*_*λi,k,q*_ is the total absorbance at the *λ*_*i*_ wavelength (nm) for a solution with a total concentration *C*_*T,k*_ (mol L^−1^) at a temperature *T*_*q*_ (K); *A*_*λi,k,q,j*_, *ε*_*λi,j*_ and *C*_*k,q,j*_ are the absorbance, molar attenuation coefficient (L mol^−1^ cm^−1^) and concentration (mol L^−1^), respectively, of species *j* in solution at the same wavelength, total concentration and temperature; *L* is the path length (cm) and *s* stands for the number of different species in solution (*i.e.*, monomer, dimer and/or higher order aggregates). *I*_*F*_ is an instrumental factor that ensures that the molar attenuation factors evaluated with a given solution are independent of the cuvette used for the evaluation. In this work, a unitary instrumental factor was established for the cuvette with a 1-cm path length, which was safely used below light absorption saturation for all the solutions over 1 ppm concentration (see Supplementary Information file). By analysing a 1.5 ppm solutions in both cuvettes (1 and 5 cm path length), a value for *I*_*F*_ of 1.056 was found for the 5 cm path length cuvette, which was used for the solutions at *C*_*T*_ ≤ 1 ppm. We consider the aggregated system as being formed by a discrete number of aggregate species, whose concentrations depend on the total ibuprofen concentration. This picture lies in between the most accepted polydisperse system, with aggregates of all sizes and compositions [50], and the system formed by monomers and aggregates (micelles) of a fixed size (*quasi-chemical* approximation [48]). In any case, all these situations can be well dealt with by the mass action law [48]. As the molar concentrations of the aqueous ibuprofen solutions are always very low (below 10^−4^ M), we can assume that the activity coefficients take the value of one. Thus, the equilibrium aggregation constants at a *T*_*q*_ temperature can be expressed as:

(2) $$K_{q,j}=\left(e^{\frac{\Delta S_{j}^{0}}{R}}\right)\left(e^{\frac{-\Delta H_{j}^{0}}{RT_{q}}}\right)=\frac{C_{k,q,j}}{C_{k,q,1}^{n_{j}}};\;2\leq j\leq s$$

where *ΔS*^*0*^_*j*_ and *ΔH*^*0*^_*j*_ stand for the standard entropy (J mol^−1^ K^−1^) and enthalpy (J mol^−1^) changes in the formation of aggregate *j* from the smallest species (*j* = *1*). The total concentration in *monomer-in-aggregate* mol-basis can be therefore evaluated as:

(3) $$C_{T,k}=n_{1}C_{k,q,1}+\sum_{j=2}^{s}n_{1}n_{j}C_{k,q,j}=n_{1}C_{k,q,1}\left(1+\sum_{j=2}^{s}n_{j}K_{q,j}C_{k,q,1}^{n_{j}-1}\right)$$

where *n*_*j*_ is the number of monomers in the species *j*. The question is how to develop an algorithm that allows the discrete aggregate orders (*n*_*1*_ *and n*_1_ × *n*_*j*_) and the related equilibrium aggregation constants to be determined from the UV absorbance data. The unknowns in equations (2), (3) are *ΔS*^*0*^_*j*_, *ΔH*^*0*^_*j*_ and *n*_*j*_, which amount to 3 × *s-*3 unknowns, since the value of *n*_*1*_ is chosen to be either 1 (monomer) or 2 (dimer), after the considerations that will be made later. These unknowns apart, for each of the *wl* values of *λ*_*i*_ wavelengths that form the absorption spectrum, *s* molar attenuation coefficients, *ε*_*λi,j*_ [1 ≤ *j* ≤ *s*; equation (1)], must be determined. Therefore, the error minimization algorithm must be able to evaluate *s* × (3 + *wl*)-3 unknowns. As commented above, when dealing with very high values of *n*_*j*_, the values of *K*_*q,j*_, *ε*_*λi,j*_ and *C*_*k,1*_^*nj*^ become unmanageable for standard calculation programs such as Microsoft Excel®. A way to tackle with this issue is to transform equations (1), (2) into:

(4) $$\frac{A_{\lambda_{i},k,q}}{I_{F}L}=n_{1}\left[\left\{\frac{\varepsilon_{\lambda_{i},1}}{n_{1}}\right\}C_{k,q,1}+\sum_{j=2}^{s}\left(\left\{n_{j}\right\}\left\{\frac{\varepsilon_{\lambda_{i},j}}{n_{1}n_{j}}\right\}e^{\left\{n_{j}\right\}\left(\left\{\frac{\Delta S_{j}^{0}}{n_{j}R}\right\}-\left\{\frac{\Delta H_{j}^{0}}{n_{j}RT_{q}}\right\}+\ln\;C_{k,q,1}\right)}\right)\right]$$

In this equation all the unknowns are indicated in curly brackets. Apart from the aggregate orders, the rest of unknowns are now expressed as normalised attenuation coefficients and normalised thermodynamic parameters. Such normalization permits a much easier error convergence to be attained. The absolute error to be minimized at each value of wavelength and total concentration values is defined as:

(5) $$E_{\lambda_{i},k,q}={\left[\frac{A_{\lambda_{i},k,q}}{I_{F}L}-\frac{A_{\lambda_{i},k,q}^{\exp}}{I_{F}L}\right]}^{2}$$

where *A*_*λi,k,q*_^*exp*^ is the experimental value of absorbance at *λ*_*i*_ and *A*_*λi,k,q*_ is evaluated via equation (4).

In this work, up to 14 solutions with increasing ibuprofen concentrations (0.1 ppm ≤ *C*_*T,k*_ ≤ 20.1 ppm) were analysed at three different temperatures (*T*_*1*_ = 20 °C, *T*_*2*_ = 30 °C and *T*_*3*_ = 40 °C). Thus, when the minimization procedure was performed with absorbance values from solutions prepared at a number *c* of concentration values (*c* ≤ 14), each analysed at a number *t* of temperature values (t ≤ 3), the total absolute error was expressed as:

(6) $$E_{T}=\frac{\sum_{q=1}^{t}\sum_{k=1}^{c}\sum_{i=1}^{wl}E_{\lambda_{i},k,q}}{t\cdot c\cdot wl}$$

Microsoft Excel® macros that make use of the Solver tool were designed with the aim of minimizing the *E*_*T*_ value. As the Solver tool can cope with a limited number of variables, only twenty wavelengths (*wl* = 20) were used in order to evaluate the thermodynamic and aggregation parameters, as well as the corresponding molar attenuation coefficients. The selected wavelengths were: 191, 192, 194, 196, 198, 200, 202, 204, 206, 208, 210, 212, 214, 216, 218, 220, 222, 224, 226 and 228 nm. Afterwards, the thermodynamic and aggregation parameters so obtained were used to determine the rest of molar attenuation coefficients in the 190–300 nm wavelength range. *C*_*k,q,1*_ is needed in order to evaluate *A*_*λi,k,q*_ by equation (4), but unfortunately it cannot be cleared from equation (3). Thus, a *user defined function* (UDF) was created in Excel® to evaluate *C*_*k,q,1*_ for every concentration value and temperature at each Solver iteration by a modified Newton-Raphson algorithm that minimizes the value of the following error function:

(7) $$E_{c}={\left[1-n_{1}\frac{C_{k,q,1}}{C_{T,k}}\left(1+\sum_{j=2}^{s}n_{j}K_{q,j}C_{k,q,1}^{n_{j}-1}\right)\right]}^{2}$$

Again, to meet the numerical limits imposed by the Excel® application, equation (7) was rearranged as follows:

(8) $$E_{c}={\left[1-n_{1}\frac{C_{k,q,1}}{C_{T,k}}\left(1+\sum_{j=2}^{s}e^{\ln\;K_{q,j}+\left(n_{j}-1\right)\ln\;C_{k,q,1}+\ln\;n_{j}}\right)\right]}^{2}$$

The modified Newton-Raphson algorithm used in the UDF to minimize *E*_*c*_ via equation (8) is described in the Supplementary Information (SI) document. Once come to this point, it goes without saying that the minimization of *E*_*T*_ [equation (6)] was an almost impossible task when the entire set of unknowns were attempted to be simultaneously evaluated for the whole concentration and temperature ranges. The number of aggregates, together with their corresponding thermodynamic parameters and orders, were all completely unknown a priori, thus preventing initial estimates. However, in this work, as the number of aggregates needed to fit all the absorbance curves was relatively low, a simple sequential fitting at increasing concentration ranges was enough to find the minimum *E*_*T*_ error. For more complex systems, we have devised a simple strategy to evaluate the number of UV-light absorbing species as a function of the total concentration that is fully explained in the Supplementary Information (SI) document.

## 4 Discussion of results

### 4.1 Dimer or monomer?

**Fig. 1:**
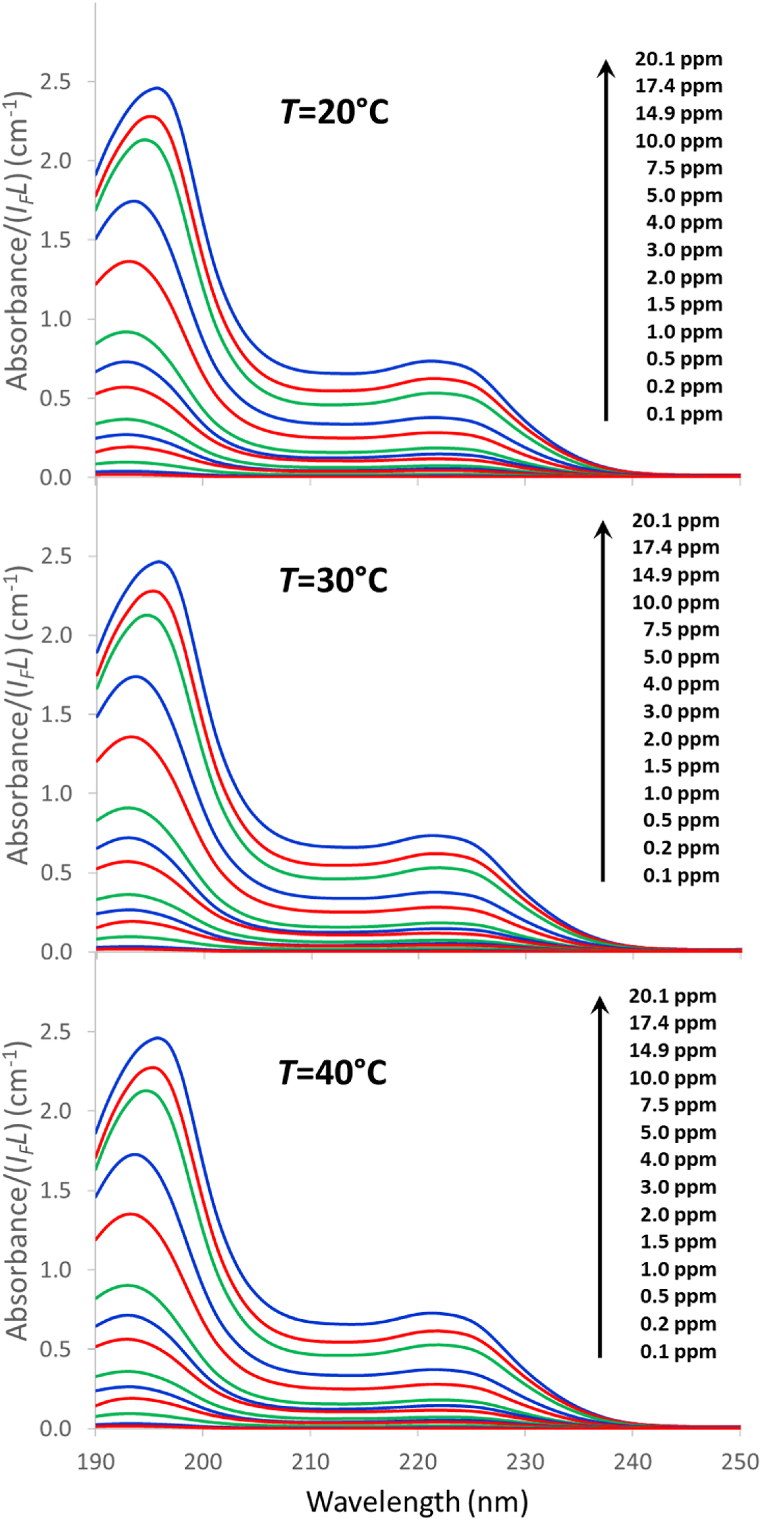
UV light absorption spectra of Ibu-H solutions at different concentrations (0.1–20.1 ppm) and temperatures (20–40 °C).

Fig. 1 shows the UV-light absorption spectra obtained for the different ibuprofen solutions prepared in this work. The main features are comprised within the 190–250 nm range. Although the 190–200 nm range is not usually considered in most works dealing with UV light absorption, we found a remarkable repeatability and consistency for the analyses performed in this work, in both the spectra and the baselines, so that we decided to include this range in the calculations. Two conspicuous peaks are seen in the spectra. The bathochromic shift of the first peak (190–200 nm range) as the ibuprofen concentration is increased reveals the concomitant appearance of different species. The second peak in the 220–230 nm range is ascribed to π-π* electronic transitions of the benzene ring [51,52]. The variation of temperature produced a negligible effect in the shape of the absorbance curves. The linear correlation between the total concentration and the absorbance in the whole concentration range studied (0.1–20.1 ppm) allowed coefficient of determination values over 0.999 to be found for any wavelength in the 219–230 nm range, with the highest value being obtained at *λ* = 225 nm (0.9994, averaged for the three temperatures tested). These results are consistent with those obtained by other authors, either for aqueous solutions or for solutions in which methanol or methanol/acetonitrile (ACN) mixtures are used either as solvent or as co-solvent [38,[53], [54], [55]]. However, whereas the constant of proportionality in the calibration equation, *C*_*T*_ = *β* × *A*_*225*_/*L*, for ibuprofen dissolved in methanol/ACN mixtures (0–6 ppm) at 20 °C was reported to be *β* = 23.22 mg cm L^−1^ [56], in the present work a value of 29.42 mg cm L^−1^ was obtained with the aqueous ibuprofen solutions. This huge difference cannot have an instrumental origin; instead it must be caused by different solvent-dependent light absorbing species.

**Fig. 2:**
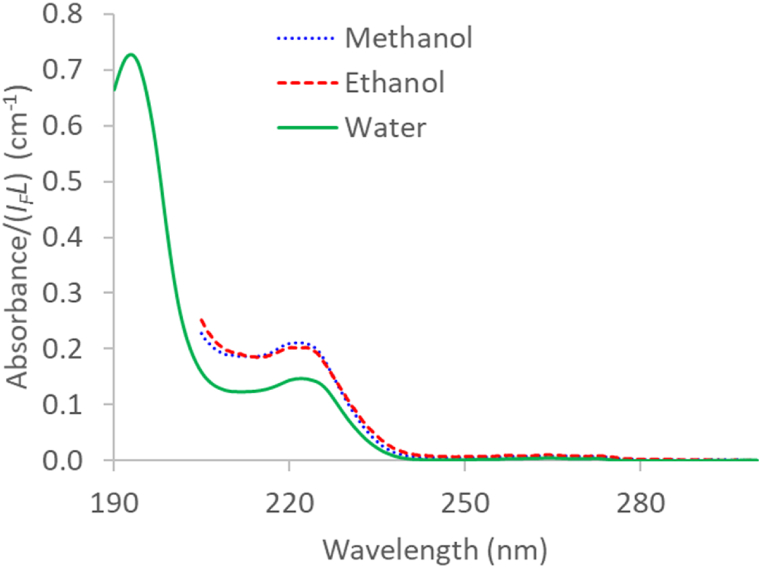
UV light absorption spectra of Ibu-H solutions (4 ppm) in different solvents (methanol, absolute ethanol and water) at 20 °C.

The dimeric ibuprofen is known to be partially converted into the monomers when ethanol is used as solvent, as confirmed by infrared and computational studies [2,6]. On the other hand, molecular dynamics simulations performed by Zhang et al. [6] revealed that the strength of hydrogen bonds in ibuprofen-water mixtures attenuated gradually in the following order: ibuprofen-ibuprofen > ibuprofen-water > water-water, suggesting that the dimer prevails over the monomer in aqueous solutions of Ibu-H. To check this, 4 ppm ibuprofen solutions were prepared with different solvents (methanol, absolute ethanol and water) and analysed after filling the reference cell with the corresponding solvent. The results are shown in Fig. 2 (205 nm cut-off wavelength for the alcoholic solutions). The light absorbing species in the aqueous solution is clearly different from that of the alcoholic solutions. The absorbance at 225 nm is also remarkably higher for the alcoholic solutions, in agreement with the *β* values described above. We believe that the different solvent polarity values are not behind the differences in the spectra shown in Fig. 2, since the π-π* electronic transitions of the benzene ring are known to be independent of the solvent polarity, at least for other similar NSAIDs such as Naproxen sodium [57]. Furthermore, differences in solvent polarity usually involve solvatochromic shifts in the UV–Visible spectra of the dissolved molecules, due to changes in charge transfer between charged ligands [[58], [59], [60]]. As can be observed in Fig. 2, the ibuprofen spectra are not affected by solvatochromism in the 215–230 nm region, with maximum absorbance values at 222 nm, regardless the solvent type. As will be seen later, ibuprofen acid in the aqueous solutions exists as a single species within the 0–6 ppm range. We can conclude that the dimer is the prevailing species in diluted aqueous solutions, whereas the UV–Vis spectra of ibuprofen in the alcoholic solutions are produced by the monomer.

### 4.2 Results of the regression analysis

**Fig. 3:**
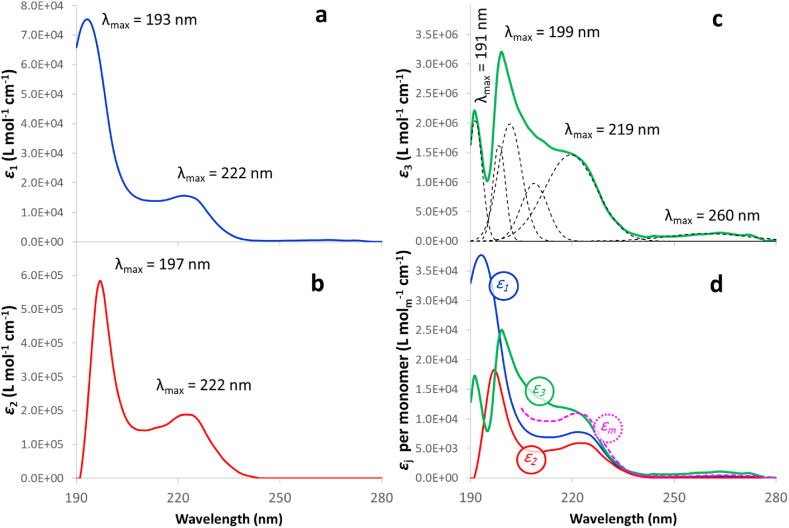
Molar attenuation coefficients for the three species (a: dimer, b: 32-monomers aggregate and c: 128-monomers aggregate) in the 190–280 nm wavelength range. Fig. 3d: Molar attenuation coefficients per mol of monomer in the aggregates and hypothetic molar attenuation coefficients of the monomer (dashed line) obtained from a 4 ppm ibuprofen solution in methanol.

**Fig. 4:**
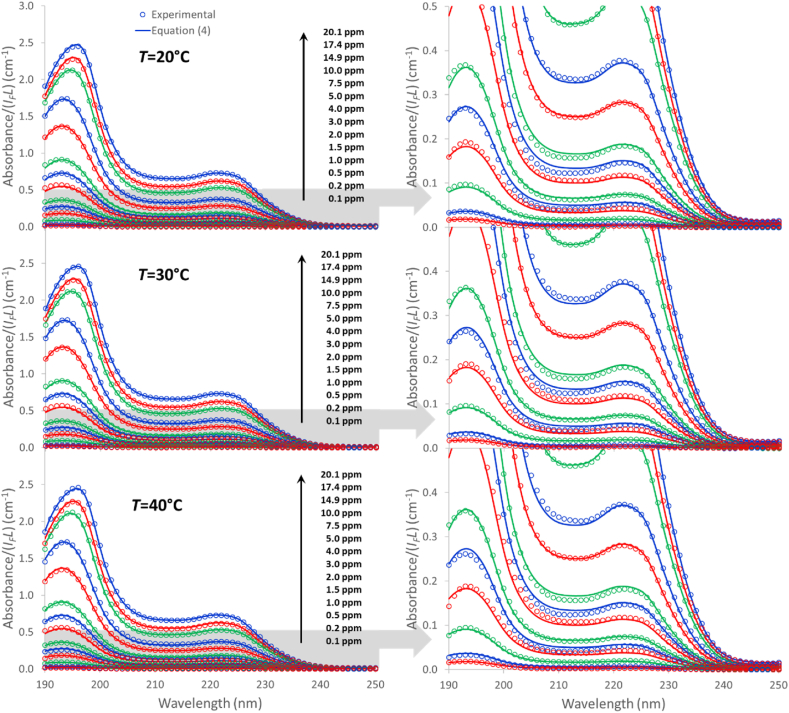
Experimental and calculated absorbance curves.

In addition to the dimer (*n*_*1*_ = 2), two aggregates formed by 32 and 128 monomeric units (*n*_*2*_ = 16 and *n*_*3*_ = 64) were found to produce the lowest value for the *E*_*T*_ error. A greater number of aggregates did not yield a significant reduction in the error, whereas it did produce evident inconsistencies in the values of the molar attenuation coefficients. Table 1 shows the aggregate orders and thermodynamic parameters of formation of all the aggregates, whereas Fig. 3a to c displays the molar attenuation coefficients for the three species in the 190–280 nm wavelength range, whose values are tabulated in the Supplementary Information file. In order to compare the specific absorption capacity of the aggregates per monomer unit, the molar attenuation coefficients were divided by the number of monomeric units for each aggregate and plotted in Fig. 3d. The molar attenuation coefficients for the monomer (*ε*_*m*_) included in Fig. 3d were evaluated from the Ibu-H/methanol spectrum plotted in Fig. 2. The features of the light absorption spectra for the different species (Fig. 3) have clear differences, which are more conspicuous in the lowest wavelength range, though all species share a maximum at ∼222 nm that corresponds to π-π* electronic transitions of the benzene rings [51,52], as could not have been otherwise. The main features are indicated in the figure, expressed as the wavelengths of the different maxima. In Fig. 3c, corresponding to the most aggregated species (*ε*_*3*_), the maximum at 219 nm is in fact a shoulder of the curve, but its position was evaluated by Gaussian deconvolution (dashed curves in the plot). All data together allowed the absorbance spectra to be calculated with a high accuracy, as can be seen in Fig. 4. The total absolute error in the 190–230 nm range was *E*_*T*_ = 6.8 × 10^−5^ ± 9.3 × 10^−5^. The right plots in Fig. 4 permit to visualize the accuracy of the model predictions for the diluted solutions. The molar fraction of each species, expressed in *monomer-in-aggregate* mol-basis, can be evaluated as:

(9) $$X_{k,q,1}=\frac{n_{1}C_{k,q,1}}{C_{T,k}}\;;\;\;\;X_{k,q,j\neq 1}=\frac{n_{1}n_{j}C_{k,q,j}}{C_{T,k}}$$

**Table 1:** Number of monomeric units (*n*_*1*_ × *n*_*j*_) and thermodynamic parameters of aggregates.

*j*	*n*_*1*_ × *n*_*j*_ (a)	*ΔS*^*0*^_*j*_ (b) (J mol^−1^ K^−1^)	*ΔH*^*0*^_*j*_ (b) (kJ mol^−1^)	*ΔS*^*0*^_*j*_ (c) (J mol_m_^−1^ K^−1^)	*ΔH*^*0*^_*j*_ (c) (kJ mol_m_^−1^)	*ΔG*^*0*^_*j*_ (c)* (kJ mol_m_^−1^)	$100\frac{\left|\Delta H_{j}^{0}\right|}{\left|\Delta H_{j}^{0}\right|+\left|T\Delta S_{j}^{0}\right|}$ *
2	32	1318.6	9.0	41.2	0.3	−11.8	2.3 %
3	128	5628.1	45.7	44.0	0.4	−12.5	2.7 %

(a) *n*_*1*_ = 2; (b) on an aggregate mol-basis; (c) on a *monomer-in-aggregate* mol-basis (mol_m_); *T = 20 °C.

**Fig. 5:**
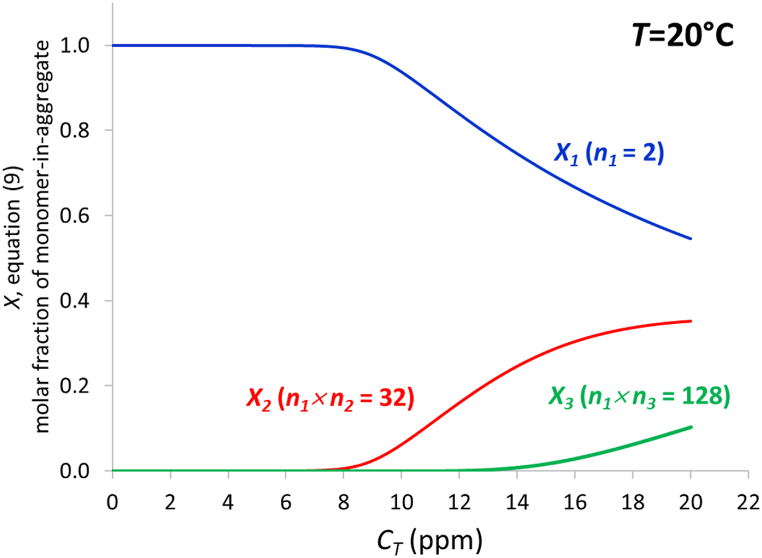
Evolution with the total ibuprofen concentration of the molar fraction of the different species at 20 °C (in brackets: number of monomeric units per aggregate).

Fig. 5 shows the evolution with the total ibuprofen concentration of the molar fraction of the different species at 20 °C, evaluated via equation (9). As can be observed in the figure, the first aggregate (16 dimers) starts to make its appearance at an ibuprofen concentration of ∼7–8 ppm, whereas the most aggregated species, probably formed by the union of 4 units of the first aggregate, appears at concentrations of 12–13 ppm.

### 4.3 Calibration equation

The universal calibration equation for a solution with *s* different species is expressed as [49]:

(10) $$C_{T,k}=\sum_{i=1}^{s}\left(\beta_{i}\frac{A_{\lambda_{i}^{ss},k,q}^{\exp}}{I_{F}L}\right)$$

**Fig. 6:**
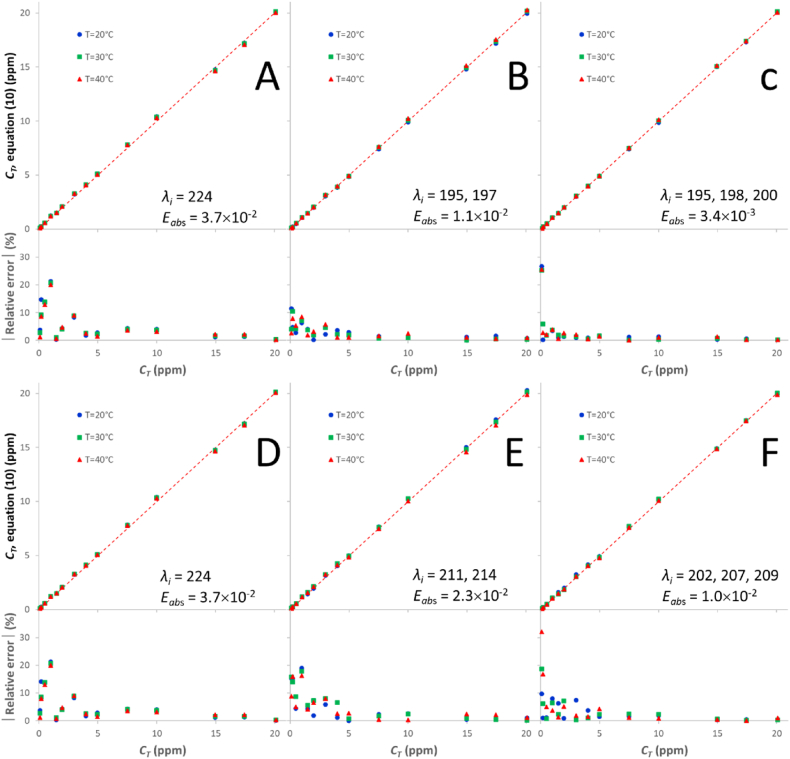
Best calibration curves and modulus of the relative error in the 0.1–20.1 ppm concentration range, obtained in different wavelength ranges (a–c: 191–230 nm; d–f: 200–230 nm) and with different numbers of species (a, d: *s* = 1; b, e: *s* = 2; c, f: *s* = 3) via equation (10).

**Fig. 7:**
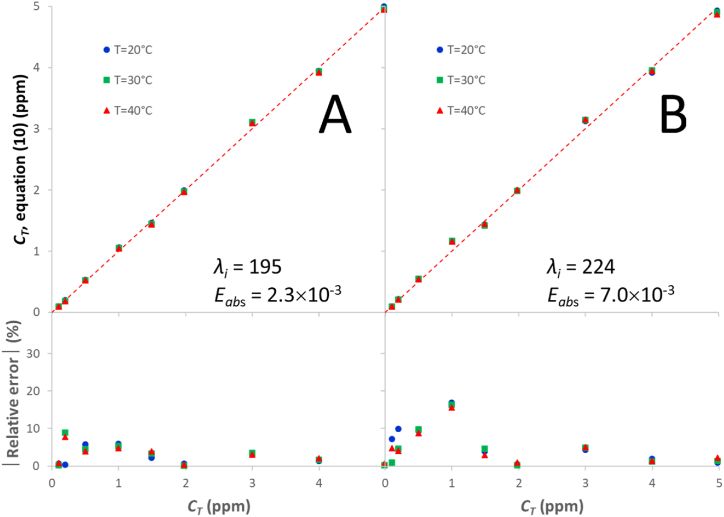
Best calibration curves and modulus of the relative error in the 0.1–5.0 ppm concentration range, obtained in different wavelength ranges (a: 191–230 nm; b: 200–230 nm) for *s* = 1 via equation (10).

Here, the *β*_*i*_ proportionality coefficients only depend on the values of the molar attenuation coefficients and the discrete aggregate orders (*n*_*j*_ values) and are independent of other variables such as the temperature, the concentration and aggregation degree, or the ionic strength [49]. In equation (10), the superscript *ss* in the *λ*_*i*_ counter refers to any given subset of *s* wavelength values without repetition in the set of *wl* values that define the whole wavelength range [1 ≤ *ss* ≤ $\left(\begin{array}{c} wl\\ s \end{array}\right)$]. Equation (10) was solved in the whole concentration and temperature ranges (0.1–20.1 ppm, 20–40 °C) for the *ss* subsets of *s* wavelengths in the 191–230 nm (*wl* = 40) and 200–230 nm (*wl* = 31) ranges. The latter was explored because it is the preferred range by most authors, who consider the range below 200 nm unreliable. Fig. 6 shows the results for the subsets that yield the minimum absolute error (*E*_*abs*_; evaluated as the average quadratic differences of the experimental and calculated concentrations) using both ranges (Fig. 6a–c: 191–230 nm; Fig. 6d–f: 200–230 nm). As the presence of three different species in the most concentrated solutions has been confirmed in this work, the logical calibration should be that performed for *s* = 3 (Fig. 6c and f). Nevertheless, Fig. 6 also shows the wavelengths and absolute errors by assuming one species (*s* = 1; Fig. 6a and d) and two species (*s* = 2; Fig. 6b and d). In all cases, the regression coefficients are over 0.999. The bottom plot in each subfigure shows the modulus of relative error in the concentration obtained with the calibration equation for each ibuprofen solution. The relative error always tends to be higher for lower concentrations, due in part to the variation of the instrumental relative error with concentration [equation (SI-15)]. As expected, the lowest absolute and relative errors are obtained when three wavelengths are used (Fig. 6c and f). The best wavelength combinations to use are 195, 198 and 200 nm (191–230 nm range) or 202, 207 and 209 nm (200–230 nm range), with noticeably better results being obtained with the first combination. As can be observed in Fig. 5, for Ibu-H concentrations below ∼7 ppm, only one species is present in solution and, therefore, only one wavelength should suffice for equation (10). Fig. 7 shows the results for this concentration range (Fig. 7a: 191–230 nm range; Fig. 7b: 200–230 nm range). Again, the best calibration is obtained when using the 191–230 nm range, with an optimal wavelength of 195 nm. For the 200–230 nm range, the 224 nm wavelength yields the lowest error. In conclusion, Fig. 6, Fig. 7 allow the calibration method to be selected as a function of the wavelength range employed in the analyses, the working concentration range and the degree of reluctance of the user to evaluate more than one linear coefficient. When a high accuracy is not a requisite, it is advisable to use a single coefficient with the absorbance values taken at 224 nm.

### 4.4 Thermodynamic parameters

All the thermodynamic parameters, expressed in *monomer-in aggregate* mol-basis, were found to be very similar (Table 1). The negative free energy values imply that aggregation is a spontaneous process, regardless the value of *n*_*j*_, whereas the positive values of enthalpy and entropy, together with the low contribution of enthalpy to the aggregation process (last column of Table 1), indicate that it is also an entropy-driven process, typical of the self-assembly of amphiphiles in water [61]. According to some authors [62,63], the essence of the entropy-driven process is the hydrophobic interaction [64,65], especially for purely hydrophobic molecules or for amphiphilic block copolymers in which the hydrophilic block is very small [66]. An ibuprofen dimer disrupts the hydrogen bonding network in water, so the water molecules rearrange around this aggregate with a corresponding loss in entropy. This loss in entropy is reduced by the aggregation of the ibuprofen dimers [66].

### 4.5 *pKa* and critical micelle concentration (*cmc*)

**Fig. 8:**
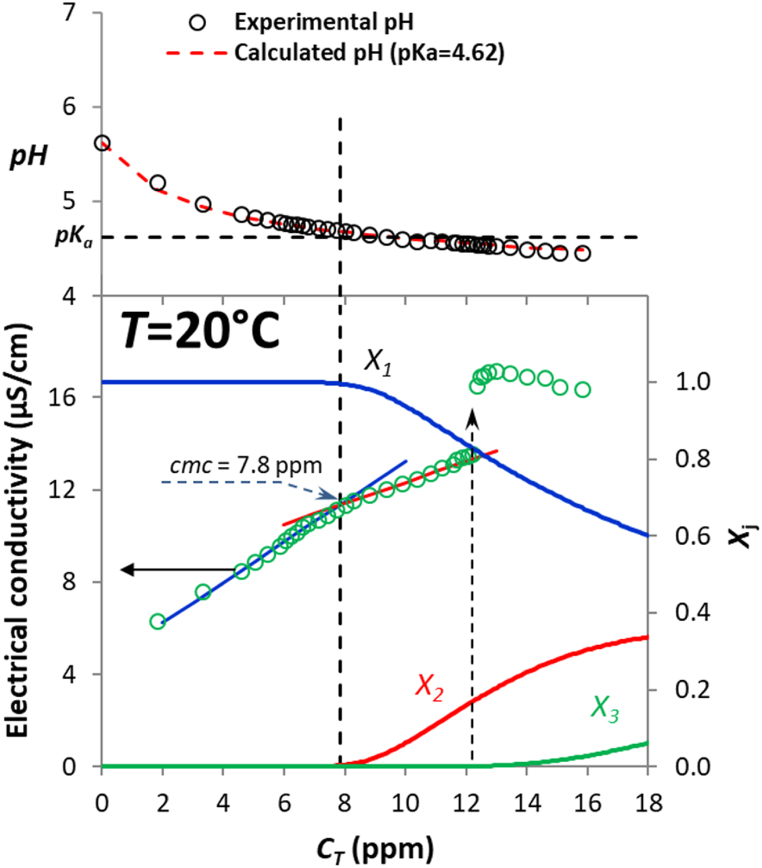
Values of experimental pH (symbols in upper plot), pH calculated via equation (11) (dashed curve in upper plot), electrical conductivity (symbols in lower plot) and molar fractions of the different species (*X*_*j*_ in lower plot) of ibuprofen solutions at 20 °C as a function of the total ibuprofen concentration.

Critical micelle concentration (*cmc*) is one of the most important characteristics of surfactants, and it is usually determined by experimental methods in which a major change in the properties of a system, such as conductance, is observed in the vicinity of *cmc*. This change occurs, as a rule, in a quite narrow region of surfactant overall concentration [67]. The electrical conductivity and pH of ibuprofen solutions in water at 20 °C were evaluated by the addition method as described in the Experimental section. The pH of the deionized water used was 5.62 (point at *C*_T_ = 0 in upper plot of Fig. 8), which implies an aqueous CO_2_ concentration of 1.35 × 10^−5^ M if the effect on pH of the HCO_3_^−^ – CO_3_^=^ equilibrium is neglected. This value is reasonable because it is just somewhat below the value expected by the Henry's law and the CO_2_ partial pressure in the lab atmosphere. With this concentration, we can determine the evolution of pH with *C*_*T*_ by means of the following third order equation:

(11) $${\left[H^{+}\right]}^{3}+K_{a}{\left[H^{+}\right]}^{2}-\left(K_{a}C_{T}+K_{a,{CO}_{2}}\left[{CO}_{2}\right]+K_{w}\right)\left[H^{+}\right]-K_{a}\left(K_{a,{CO}_{2}}\left[{CO}_{2}\right]+K_{w}\right)=0$$

in which the effect of ibuprofen aggregation on pH is ignored (all monomers are assumed to have the same *K*_*a*_ value, whether they are aggregated or not). In equation (11), the first acidity constant of CO_2_ in water (*K*_*a,CO2*_) takes the value of 4.42 × 10^−7^ [68] and *K*_*w*_ is the ion product of water. The best fitting of equation (11) to the experimental values of pH was obtained for a *pK*_*a*_ value of 4.62 (top plot on Fig. 8). This value is within the range of published *pK*_*a*_ values for ibuprofen in water (4.40 [69], 4.51 [70], 4.59 [71], 4.61 [72], 4.47–4.70 [73]).

The bottom plot in Fig. 8 shows the electrical conductivity values of ibuprofen solutions at 20 °C as a function of the total ibuprofen concentration together with the expected molar fractions of the different species (Fig. 5). A first change in the slope of the conductivity-concentration trend was determined at 7.8 ppm, a concentration that should therefore be considered the critical micelle concentration (*cmc*) for ibuprofen acid in deionized water. As can be observed, the *cmc* value clearly represents the zone where the dimer starts to condense as a 32-monomer units aggregate. Furthermore, the pH at the critical micelle concentration point is close to the *pK*_*a*_ value (Fig. 8), suggesting that agglomeration starts to occur when the pH value is below the *pK*_*a*_. From equilibrium considerations, at pH values below the *pK*_*a*_ the neutral form of ibuprofen is favoured over the anionic form [74]; this implies a diminution of the conductivity (smaller fraction of charged species) and the parallel appearance of agglomeration (smaller electrical repulsive forces). An abrupt increment in conductivity is also observed at *C*_*T*_ ∼12.5 ppm, followed by a decrease in conductivity at higher concentrations. The sudden jump in electrical conductivity over a very short range of concentration, which was confirmed in repeated tests, coincides with the beginning of the formation of the 128-monomer units aggregate (Fig. 8). We have no explanation for this phenomenon, though a similar conductivity jump was reported in a work by Modaressi et al. [75] on the aggregation of imidazolium ionic liquids in aqueous solutions. As in the present work, the jump occurred at a concentration higher than the *cmc*. These authors were also unable to offer an explanation for the jump, and simply said: “*We suppose that the jump arises as a consequence of a change in the aggregate organisation and solvation and is directly induced by interactions between aggregates*” [75]. In fact, we can confirm that the onset of a new aggregation process coincides with the jump (Fig. 8), so that we fully agree with the hypothesis of Modaressi et al. [75].

### 4.6 Kinetics of aqueous Ibu-H dissolution

**Fig. 9:**
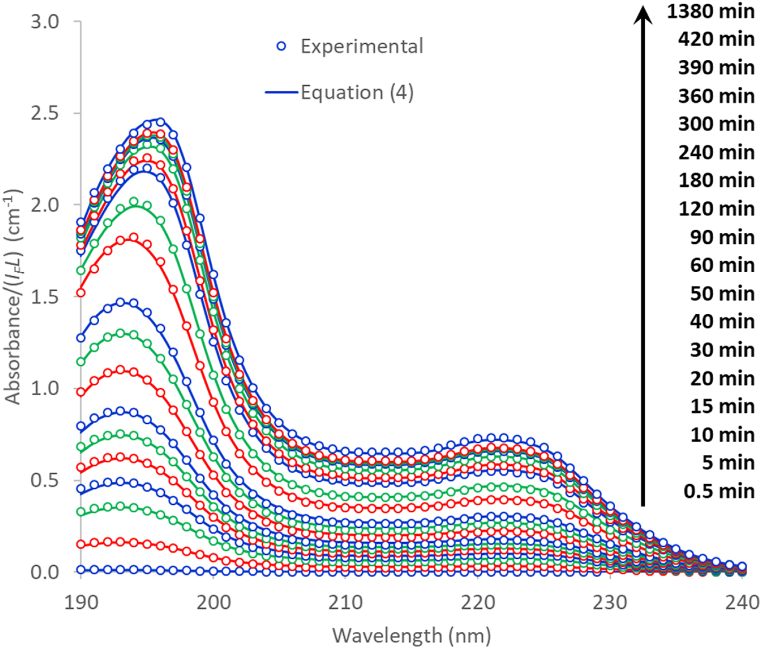
Experimental and calculated absorbance spectra of a just-prepared 20 ppm ibuprofen solution at 20 °C and different stirring times.

**Fig. 10:**
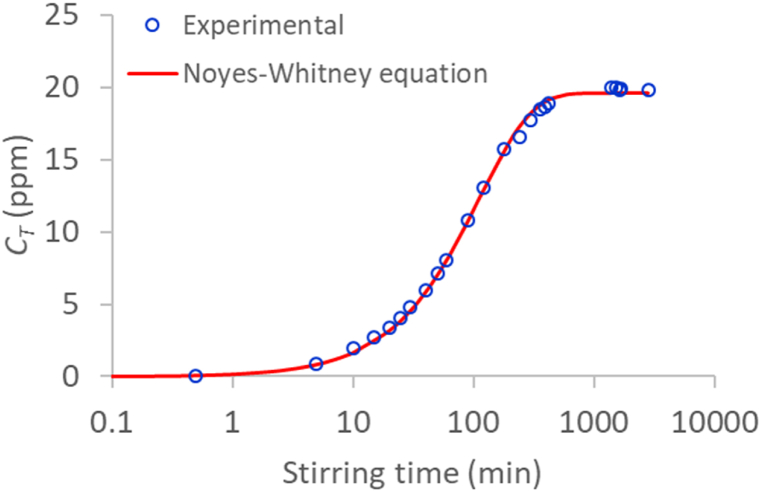
Evolution of *C*_*T*_ (evaluated from the spectra in Fig. 9) with the stirring time for a just-prepared 20 ppm ibuprofen solution at 20 °C. The dashed line represents the fitting of the Noyes-Whitney first order equation to the *C*_*T*_ values.

Fig. 9 shows the absorbance spectra of a just-prepared 20 ppm ibuprofen solution at 20 °C and different stirring times. With the data from Table 1 and Fig. 3, a simple least-squares fit made it possible to calculate the actual Ibu-H concentration (*C*_*T*_) corresponding to each of the stirring times. The absorbance spectra evaluated by equation (4) for the so-obtained *C*_*T*_ values are also shown in Fig. 9. The goodness of fit observed in the figure implies that the aggregation equilibria are reached very much faster than the dissolution equilibrium. Fig. 10 shows the evolution of the evaluated *C*_*T*_ values with the stirring time. The classical Noyes-Whitney first order equation [76], equation (12), was found to be correctly fitted to the experimental [*C*_*T*_, *t*] values:

(12) $$\frac{dC_{T}}{dt}=k\left(C_{T}^{*}-C_{T}\right)$$

where *t* is the stirring time (min) and *C*_T_* is the Ibu-H concentration after complete dissolution. The results of the fit are shown in Fig. 10 (*k* = 8.83 × 10^−3^ min^−1^). Thus, around 23 h are needed for the complete dissolution of Ibu-H at the conditions used in this work.

## 5 Conclusions

The calculation algorithm developed in this work allowed the aggregate orders and thermodynamic parameters of all species co-existing in aqueous ibuprofen solutions (0.1–20.1 ppm, 20–40 °C) to be obtained from experimental UV light absorbance spectra. The algorithm was specifically designed in order to overcome the limits imposed by the standard spreadsheets when dealing with extremely high exponents (aggregate orders). From theoretical results reported in the literature and the comparison of the UV–Visible spectra of ibuprofen in different solvents (methanol, absolute ethanol and water) it was possible to confirm the absence of monomeric ibuprofen acid in water solutions. Three species were found to co-exist in solution at the highest concentration tested: the dimer and two more aggregates formed by 32 and 128 monomeric units. The evolution of the molar fraction of each species with the total ibuprofen concentration was evaluated. With the help of electrical conductivity tests, a critical micelle concentration of 7.8 ppm was estimated for this system. The appearance of the first aggregate (32 monomeric units) occurs when the pH drops below the *pK*_*a*_ value, which was determined to be 4.62. At higher ibuprofen concentrations, a sudden jump in the conductivity seems to be related to the onset of formation of the second aggregate (128 monomeric units). The values of the thermodynamic parameters reveal that ibuprofen aggregation is a spontaneous and entropy-driven process. In this work a varied menu of alternatives is offered with respect to the calibration curve of ibuprofen in water, though the linear calibration of ibuprofen concentration with absorbance in the 0.1–20.1 ppm range might be reasonably performed with the absorbance values obtained at 224 nm. The dissolution rate of the commercial ibuprofen used in this work, evaluated from the absorbance spectra and successfully reproduced with the Noyes-Whitney first order equation, was found to be rather slow.

## Data availability statement

Data will be made available on request.

## CRediT authorship contribution statement

**Gregorio Marbán:** CALZON, Conceptualization, Software, Writing – original draft. **Amparo Fernández-Pérez:** Formal analysis, Investigation, Writing – review & editing. **Sonia Álvarez-García:** Investigation, Writing – review & editing.

## Declaration of competing interest

The authors declare that they have no known competing financial interests or personal relationships that could have appeared to influence the work reported in this paper.
