# Supplementary material for: Ultraviolet light spectroscopic characterization of ibuprofen acid aggregation in deionized water
Source: Heliyon. 2023 Oct 20;9(11):e21260. doi: 10.1016/j.heliyon.2023.e21260 (PMC10598541; doi:10.1016/j.heliyon.2023.e21260)
Supplement: Multimedia component 1 [file mmc1.docx]

Ultraviolet light spectroscopic characterization of ibuprofen acid aggregation in deionized water

Gregorio Marbán^[[1]](#footnote-1)†^, Amparo Fernández-Pérez^†^ and Sonia Álvarez-García^‡^

^†^Instituto de Ciencia y Tecnología del Carbono (INCAR-CSIC) – c/Francisco Pintado Fe 26, 33011‑Oviedo (Spain). Tel. +34 985119090

^‡^Departamento de Ingeniería Química y Tecnología del Medio Ambiente, Facultad de Químicas, Universidad de Oviedo- c/ Julián Clavería 8, 33006-Oviedo (Spain). Tel. +34 985104243

**Supplementary Information**


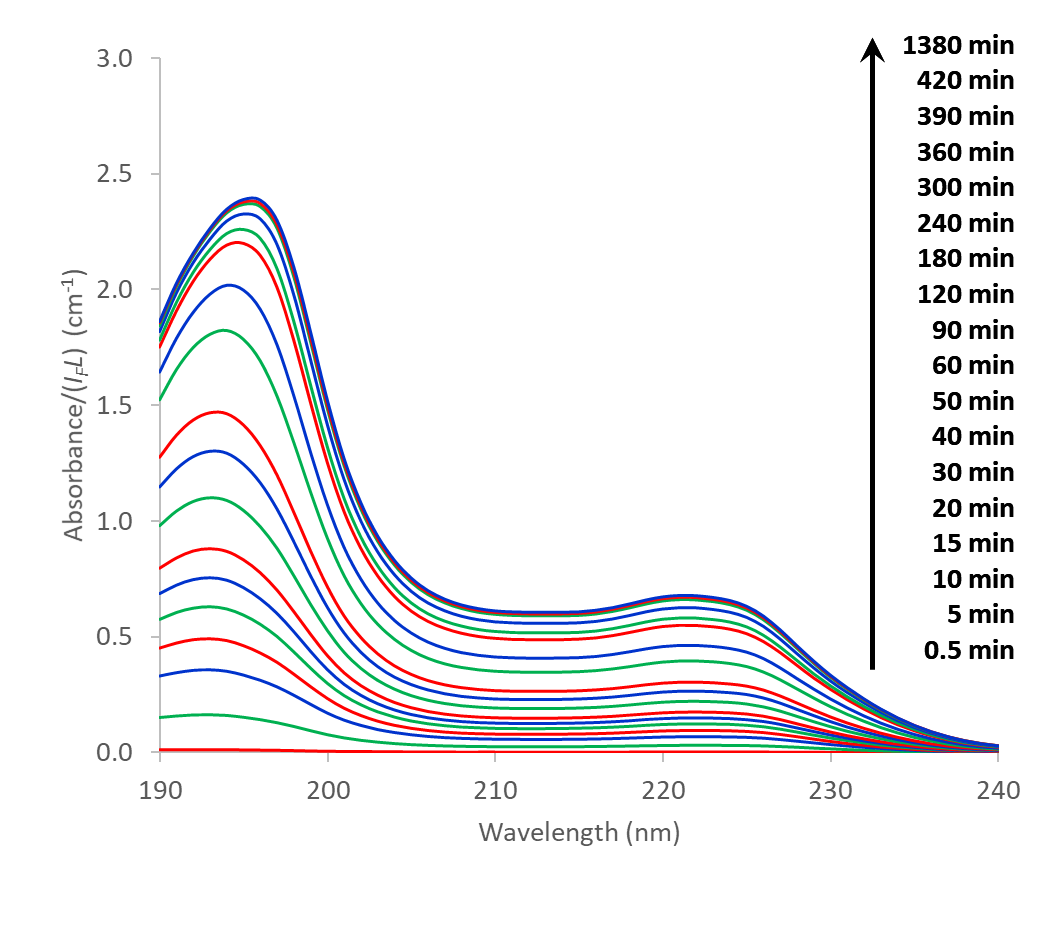


**Figure SI-1.** Absorbance spectra of a just-prepared 20 ppm ibuprofen solution at different stirring times (*T*=20°C)

Absence of saturation in light absorption for spectra in Figure SI-1

Potassium dichromate solutions in diluted sulphuric acid were used as standards to check the highest reliable absorbance for the 1‑cm path length cell [1]. Figure SI-2 shows the Absorbance/Concentration curves for absorbance values below 3.5. As can be observed, all curves nicely overlap. For values of absorbance over 3.5, saturation in light absorption yielded non‑overlapping Absorbance/C values for the different concentrations. Figure SI-2 shows the calibration curves at 256 and 350 nm, with very high regression coefficients even though they include absorbance values over 2.5. These results validate the spectra shown in Figure SI-1, in which the absorbance values were below 2.5 in all cases.


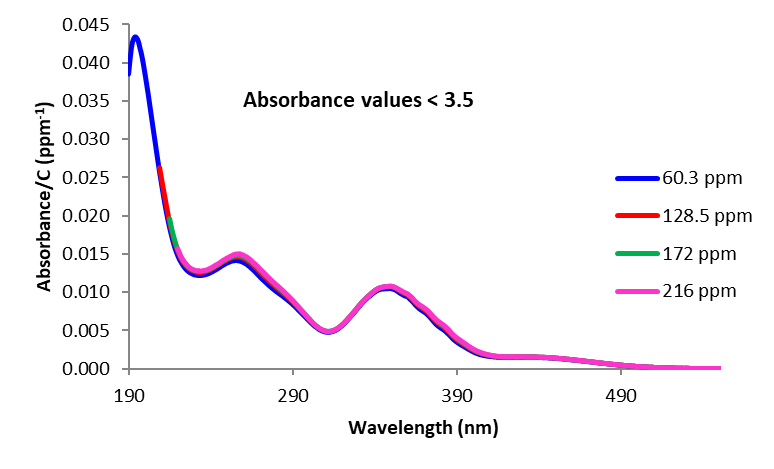


Figure SI-2. Absorbance/Concentration curves for absorbance values below 3.5


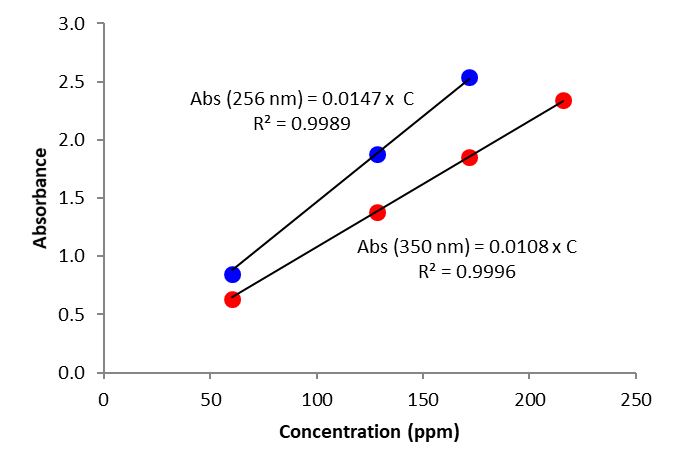


Figure SI-3. Calibration curves at 256 and 350 nm

Modified Newton-Raphson algorithm

A user defined function (UDF) was created in Excel^®^ to evaluate *C_k,q,1_* for every concentration value and temperature by a modified Newton-Raphson algorithm that minimizes the value of the following error function:

$E_{c}=\left[ 1-n_{1}\frac{C_{k,q,1}}{C_{T,k}}\left( 1+\sum_{j=2}^{s} e^{\ln K_{q,j}+\left( n_{j}-1 \right)\ln C_{k,q,1}+\ln n_{j}} \right) \right]^{2}$ (SI-1)

To minimize this error, several Newton-Raphson-based algorithms were tested, including the standard algorithm [2,3]. It was found that the best performing algorithm in the whole concentration range was one based in the numerical evaluation of the error derivative:

$C_{k,q,1}=C_{k,q,1*}-0.1\frac{E_{c}\Delta C}{\Delta E_{c}}$ (SI-2)

in which:

$C_{k,q,1*}=0.5\left( C_{k,q,1*,a}+C_{k,q,1*,b} \right)$ (SI-3)

$E_{c}=0.5\left[ E_{c}\left( C_{k,q,1*,a} \right)+E_{c}\left( C_{k,q,1*,b} \right) \right]$ (SI-4)

$\Delta E_{c}=E_{c}\left( C_{k,q,1*,a} \right)-E_{c}\left( C_{k,q,1*,b} \right)$ (SI-5)

$\Delta C=C_{k,q,1*,a}-C_{k,q,1*,b}$ (SI-6)

In the first step:

$C_{k,q,1*,a}=0.001\times C_{T,k}$ (SI-7)

$C_{k,q,1*,b}=0.0012\times C_{T,k}$ (SI-8)

whereas in successive steps:

$C_{k,q,1*,a}=C_{k,q,1}$ (SI-9)

$C_{k,q,1*,b}=C_{k,q,1}\times\left( 1+C_{k,q,1*}-C_{k,q,1} \right)$ (SI-10)

The convergence was assumed to be achieved for a relative error percentage, *E_r_*, below 10^‑6^:

$E_{r}=100\times\left| 1-\frac{C_{k,q,1}}{C_{k,q,1*}} \right|<{10}^{-6}$ (SI-11)

Evaluation of the number of UV-light absorbing species as a function of the total concentration

There is a way which is not particularly tricky that allows the number of UV-light absorbing species that are present in the different solutions to be estimated. This knowledge can be achieved via the following equation [4]:

$C_{T,k}=\sum_{i=1}^{s} \left( \beta_{i}\frac{A_{\lambda_{i}^{ss},k,q}^{exp}}{I_{F}L} \right)$ (SI-12)

Here, the *β_i_* proportionality coefficients only depend on the values of the molar attenuation coefficients and the discrete aggregate orders (*n_j_* values) and are independent of other variables such as the temperature, the concentration and aggregation degree, or the ionic strength [4]. In equation (SI-12), the superscript *ss* in the *λ_i_* counter refers to any given subset of *s* wavelength values without repetition in the set of *wl* values that define the whole wavelength range [1 ≤ *ss* ≤ $\left( \begin{matrix} wl \\ s \end{matrix} \right)$]. Within a given concentration range (1 ≤ *k* ≤ *c*), the minimum number *s* of *β_i_* coefficients that are needed to attain a sufficiently low error of the least-squares fitting of equation (SI-12) to the absorbance data, averaged for all $\left( \begin{matrix} wl \\ s \end{matrix} \right)$ wavelength combinations, is therefore equal to the number of species absorbing light in the analysed concentration range. The average absolute error for a given concentration range is evaluated as:

$E_{a}=\frac{\sum_{ss=1}^{\left( \begin{matrix} wl \\ s \end{matrix} \right)} \sum_{q=1}^{t} \sum_{k=1}^{c} \left[ \sum_{i=1}^{s} \left( \beta_{i}\frac{A_{\lambda_{i}^{ss},k,q}^{exp}}{I_{F}L} \right)-C_{T,k}^{exp} \right]^{2}}{\left( \begin{matrix} wl \\ s \end{matrix} \right)\cdot t\cdot c}$ (SI-13)

in which *C_T,k_^exp^* are the experimental concentration values. The absolute error does not permit a comparison among different concentration ranges. To do this, a weighted arithmetic mean relative error was evaluated with the sets of *β_i_* coefficients that minimize the *E_a_* value as:

$E_{w}=100\times\frac{\sum_{ss=1}^{\left( \begin{matrix} wl \\ s \end{matrix} \right)} \sum_{q=1}^{t} \sum_{k=1}^{c} w_{k}\left| \frac{\sum_{i=1}^{s} \left( \beta_{i}\frac{A_{\lambda_{i}^{ss},k,q}^{exp}}{I_{F}L} \right)}{C_{T,k}^{exp}}-1 \right|}{\left( \begin{matrix} wl \\ s \end{matrix} \right)\cdot t\cdot\sum_{k=1}^{c} w_{k}}$ (SI-14)

In this work, the non-negative weights, *w_k_*, were evaluated as the inverse of the concentration‑related relative error of the light absorption tests (instrumental relative error). The instrumental relative error was estimated by means of repeated measures with solutions at given concentrations and temperatures, as the standard deviation to the mean absorbance value ratio averaged in the 193-225 nm range (range of highest light absorption; Figure 1 in the manuscript). In this way, the following expression was obtained:

$w_{k}=2.86{10}^{5}C_{T,k}^{0.5173}$ (SI-15)

Thus, by comparing the values of *E_w_* at increasing concentration ranges for different values of *s*, a clear picture of the evolution of the number of species with the total ibuprofen concentration can be obtained. For low values of *s* (i.e., *s* ≤ 4), all wavelength combinations in a selected range can be used to minimize *E_a_*. For higher values of *s*, a fixed number of randomly distributed combinations can be employed. For a large number of light absorbing species (over 4), the precious information gained by this procedure allows the minimization of *E_T_* to be performed at increasing values of *c* (increasing *C_T_* ranges) with previously selected values of *s*, so that the attenuation coefficients, aggregate orders and thermodynamic parameters of formation might be sequentially determined and refined. Due to the low number of light absorbing species in the system under study, there was no need to put into practice this evaluation procedure in the present work. It is reported here because it can be useful for more complex systems.

Molar attenuation coefficients of monomer and aggregates

| ***λ_i_* (nm)** | **Molar attenuation coefficients (L mol^‑1^ cm^‑1^)** | | | |
| --- | --- | --- | --- | --- |
|  | ***n_1_*=2** | ***n_1_×n_2_*=32** | ***n_1_×n_3_*=128** | ***Monomer**** |
| 190 | 65850.1 | 0.0 | 1650007.3 | - |
| 191 | 70933.6 | 0.0 | 2198444.8 | - |
| 192 | 74091.8 | 71375.8 | 2023172.5 | - |
| 193 | 75353.4 | 178703.2 | 1631808.3 | - |
| 194 | 74696.4 | 311458.4 | 1188433.4 | - |
| 195 | 71836.7 | 442783.6 | 1016627.4 | - |
| 196 | 67161.2 | 548244.9 | 1307713.3 | - |
| 197 | 60731.3 | 584927.9 | 2108991.8 | - |
| 198 | 52817.5 | 550842.3 | 2888420.3 | - |
| 199 | 44583.4 | 473074.6 | 3201191.0 | - |
| 200 | 36947.4 | 393312.5 | 3060167.5 | - |
| 201 | 30678.2 | 319845.2 | 2835368.8 | - |
| 202 | 25996.9 | 263213.5 | 2620351.6 | - |
| 203 | 22475.9 | 222396.6 | 2408235.7 | - |
| 204 | 19814.8 | 192058.4 | 2242069.4 | - |
| 205 | 17895.7 | 170121.6 | 2126890.4 | 11758.5 |
| 206 | 16491.8 | 156118.5 | 2021375.6 | 10984.9 |
| 207 | 15503.7 | 148591.0 | 1921250.1 | 10469.2 |
| 208 | 14815.8 | 143566.3 | 1856586.7 | 10108.2 |
| 209 | 14341.4 | 142362.7 | 1788708.9 | 9850.3 |
| 210 | 14056.5 | 142026.3 | 1741674.0 | 9695.6 |
| 211 | 13905.9 | 142129.4 | 1710985.2 | 9695.6 |
| 212 | 13815.4 | 144956.7 | 1667339.4 | 9644.1 |
| 213 | 13779.0 | 148351.2 | 1614439.6 | 9644.1 |
| 214 | 13790.4 | 150902.1 | 1575420.1 | 9592.5 |
| 215 | 13856.0 | 153122.1 | 1551888.7 | 9644.1 |
| 216 | 14026.3 | 156258.0 | 1537036.9 | 9798.8 |
| 217 | 14296.7 | 161315.4 | 1529110.8 | 10005.1 |
| 218 | 14642.8 | 168901.8 | 1514564.1 | 10314.5 |
| 219 | 15024.7 | 177113.0 | 1494948.0 | 10623.9 |
| 220 | 15368.4 | 183523.5 | 1475415.3 | 10830.2 |
| 221 | 15557.0 | 187489.4 | 1444788.5 | 10881.8 |
| 222 | 15577.2 | 188841.8 | 1403017.5 | 10881.8 |
| 223 | 15434.9 | 188486.3 | 1355225.1 | 10778.7 |
| 224 | 15152.4 | 187581.9 | 1282390.6 | 10572.4 |
| 225 | 14680.5 | 182033.9 | 1189663.8 | 10108.2 |
| 226 | 13822.0 | 170796.5 | 1068655.9 | 9283.1 |
| 227 | 12536.6 | 154815.4 | 936377.7 | 8303.2 |
| 228 | 11017.2 | 135666.5 | 807393.3 | 7271.7 |
| 229 | 9447.7 | 118270.7 | 666813.7 | 6240.3 |
| 230 | 7988.8 | 101511.1 | 555882.1 | 5312.0 |
| 231 | 6646.8 | 87565.6 | 460292.5 | 4435.2 |
| 232 | 5435.1 | 73909.1 | 385631.1 | 3610.1 |
| 233 | 4371.8 | 60338.3 | 326988.6 | 2888.1 |
| 234 | 3461.7 | 48801.2 | 271333.5 | 2269.2 |
| 235 | 2674.5 | 39758.4 | 210519.1 | 1753.5 |
| 236 | 2039.9 | 31311.1 | 170612.7 | 1289.3 |
| 237 | 1538.8 | 23671.5 | 140870.1 | 979.9 |
| 238 | 1150.8 | 16990.8 | 121870.2 | 722.0 |
| 239 | 868.9 | 12012.7 | 103787.7 | 567.3 |
| 240 | 662.9 | 8690.4 | 89679.6 | 412.6 |
| 241 | 526.2 | 6419.1 | 75789.0 | 361.0 |
| 242 | 440.5 | 4307.7 | 71635.1 | 309.4 |
| 243 | 398.5 | 2140.4 | 79091.4 | 257.9 |
| 244 | 376.6 | 23.6 | 90331.1 | 257.9 |
| 245 | 352.7 | 103.1 | 80698.1 | 257.9 |
| 246 | 329.3 | 0.0 | 79417.1 | 257.9 |
| 247 | 319.6 | 0.0 | 79012.3 | 257.9 |
| 248 | 316.1 | 0.0 | 79894.5 | 206.3 |
| 249 | 327.8 | 0.0 | 79793.1 | 257.9 |
| 250 | 349.1 | 0.0 | 82241.7 | 257.9 |
| 251 | 379.2 | 0.0 | 84581.7 | 257.9 |
| 252 | 389.6 | 0.0 | 89115.3 | 257.9 |
| 253 | 392.5 | 0.0 | 93614.2 | 309.4 |
| 254 | 410.9 | 0.0 | 96950.2 | 309.4 |
| 255 | 429.1 | 0.0 | 104364.1 | 309.4 |
| 256 | 446.0 | 0.0 | 112024.3 | 309.4 |
| 257 | 472.9 | 0.0 | 115633.3 | 361.0 |
| 258 | 491.6 | 0.0 | 118477.9 | 361.0 |
| 259 | 500.7 | 0.0 | 119376.3 | 361.0 |
| 260 | 504.7 | 0.0 | 122387.9 | 361.0 |
| 261 | 515.6 | 0.0 | 127504.8 | 361.0 |
| 262 | 538.0 | 0.0 | 136570.3 | 412.6 |
| 263 | 571.9 | 0.0 | 142580.5 | 412.6 |
| 264 | 597.5 | 0.0 | 142916.6 | 412.6 |
| 265 | 587.4 | 0.0 | 134930.8 | 412.6 |
| 266 | 551.1 | 0.0 | 129287.6 | 412.6 |
| 267 | 509.9 | 0.0 | 121194.1 | 412.6 |
| 268 | 461.3 | 0.0 | 111899.9 | 412.6 |
| 269 | 416.5 | 0.0 | 103094.0 | 361.0 |
| 270 | 393.4 | 0.0 | 99952.2 | 361.0 |
| 271 | 416.2 | 0.0 | 106030.8 | 361.0 |
| 272 | 442.9 | 0.0 | 109529.7 | 361.0 |
| 273 | 427.4 | 0.0 | 95931.5 | 361.0 |
| 274 | 333.8 | 0.0 | 73160.4 | 309.4 |
| 275 | 198.7 | 0.0 | 50387.5 | 257.9 |
| 276 | 96.4 | 0.0 | 35042.4 | 154.7 |
| 277 | 61.6 | 0.0 | 352.4 | 103.1 |
| 278 | 2.7 | 0.0 | 18996.2 | 103.1 |
| 279 | 0.0 | 0.0 | 9243.6 | 103.1 |
| 280 | 0.0 | 0.0 | 6186.7 | 51.6 |
| 281 | 0.0 | 0.0 | 3413.1 | 51.6 |
| 282 | 0.0 | 0.0 | 3153.5 | 51.6 |
| 283 | 0.0 | 0.0 | 862.2 | 51.6 |
| 284 | 0.0 | 0.0 | 2622.1 | 51.6 |
| 285 | 0.0 | 0.0 | 4711.4 | 51.6 |
| 286 | 0.0 | 0.0 | 4536.9 | 51.6 |
| 287 | 2.4 | 0.0 | 5542.1 | 51.6 |
| 288 | 12.5 | 0.0 | 4967.3 | 0.0 |
| 289 | 12.5 | 0.0 | 5075.3 | 0.0 |
| 290 | 14.8 | 0.0 | 5683.1 | 51.6 |
| 291 | 22.9 | 0.0 | 4635.8 | 0.0 |
| 292 | 25.1 | 0.0 | 4851.2 | 51.6 |
| 293 | 17.2 | 0.0 | 4212.8 | 51.6 |
| 294 | 23.7 | 0.0 | 2013.4 | 51.6 |
| 295 | 28.8 | 0.0 | 0.0 | 0.0 |
| 296 | 21.7 | 0.0 | 439.8 | -51.6 |
| 297 | 9.7 | 98.9 | 0.0 | 51.6 |
| 298 | 0.0 | 103.0 | 0.0 | 0.0 |
| 299 | 0.0 | 162.5 | 0.0 | 0.0 |
| 300 | 0.0 | 0.0 | 0.0 | 0.0 |

* Evaluated from a 4 ppm ibuprofen solution in methanol

Literature

[1] R.W. Burke, R. Mavrodineanu, Certification and use of acidic potassium dichromate solutions as an ultraviolet absorbance standard SRM 935, US Department of Commerce, National Bureau of Standards, 1977.

[2] S.M. Kang, W. Nazeer, M. Tanveer, Q. Mehmood, K. Rehman, Improvements in Newton-Rapshon method for nonlinear equations using modified Adomian decomposition method, Int. J. Math. Anal. 9 (2015) 1919–1928.

[3] B. Saheya, G. Chen, Y. Sui, C. Wu, A new Newton-like method for solving nonlinear equations, Springerplus. 5 (2016) 1269. https://doi.org/10.1186/s40064-016-2909-7.

[4] A. Fernández-Pérez, G. Marbán, Visible Light Spectroscopic Analysis of Methylene Blue in Water, J. Appl. Spectrosc. 88 (2022) 1284–1290. https://doi.org/10.1007/s10812-022-01310-y.

1. Corresponding Author: e-mail: [greca@incar.csic.es](mailto:greca@incar.csic.es) (G. Marbán) [↑](#footnote-ref-1)
